# Phase and Orientation Control of NiTiO_3_ Thin Films

**DOI:** 10.3390/ma13010112

**Published:** 2019-12-25

**Authors:** Jon Einar Bratvold, Helmer Fjellvåg, Ola Nilsen

**Affiliations:** Centre for Materials Science and Nanotechnology (SMN), Department of Chemistry, University of Oslo, P.O. Box 1033 Blindern, N-0315 Oslo, Norway

**Keywords:** metal oxide thin films, atomic layer deposition, ALD, crystallography, epitaxy, NiTiO_3_

## Abstract

Subtle changes in the atomic arrangement of NiTiO_3_ in the ilmenite structure affects its symmetry and properties. At high temperatures, the cations are randomly distributed throughout the structure, resulting in the corundum structure with *R*−3*c* symmetry. Upon cooling, the cations order in alternating layers along the crystallographic *c* axis, resulting in the ilmenite structure with *R*−3 symmetry. Related to this is the *R*3*c* symmetry, where the cations alternate both perpendicularly and along the *c* axis. NiTiO_3_ with the latter structure is highly interesting as it exhibits ferroelectric properties. The close relationship between structure and properties for ilmenite-related structures emphasizes the importance of being able to control the symmetry during synthesis. We show that the orientation and symmetry of thin films of NiTiO_3_ formed by atomic layer deposition (ALD) can be controlled by choice of substrate. The disordered phase (*R*−3*c*), previously only observed at elevated temperatures, have been deposited at 250 °C on α-Al_2_O_3_ substrates, while post-deposition annealing at moderate temperatures (650 °C) induces ordering (*R*−3). We have in addition explored the symmetry and epitaxial orientation obtained when deposited on substrates of LaAlO_3_(100), SrTiO_3_(100) and MgO(100). The presented work demonstrates the possibilities of ALD to form metastable phases through choice of substrates.

## 1. Introduction

**Figure 1:**
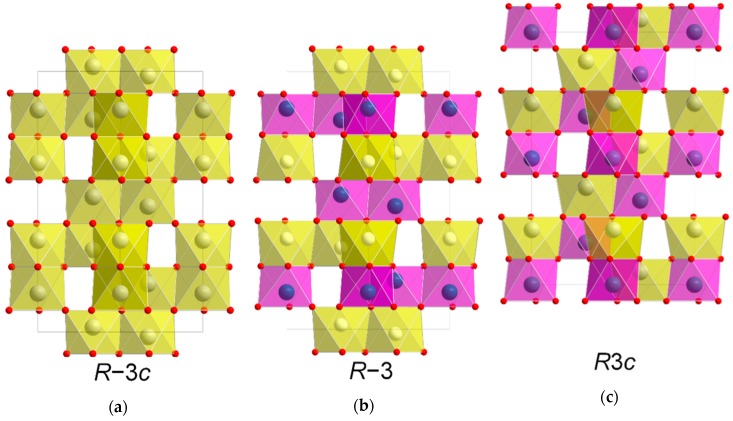
Schematic showing the similarity between the unit cells of space groups *R*−3*c* (**a**), *R*−3 (**b**) and *R*3*c* (c), viewed along [110]. The *R*3*c* structure (**c**) is shifted up one cation layer to emphasize the relation to the two other structures.

NiTiO_3_ (NTO) is a technologically relevant material proven suitable within photo catalysis, high-*k* dielectrics and non-volatile memory [1,2,3,4,5]. In addition, ab initio calculations predict ferroelectricity and crystal structure dependent weak ferromagnetism, potentially suitable for sensor, microwave and spintronic applications [6]. At elevated temperatures, NTO adopts the corundum structure (space group *R*−3*c*, Figure 1a) with random distribution of cations [7]. This disordered phase has so far been unquenchable, as cooling induces ordering of the two different cations in alternating layers perpendicular to the crystallographic *c* axis. As ordering occurs the *c* glide plane is lost, and the structure is known as the ilmenite structure (space group *R*−3, Figure 1b) [8]. This is also the case for ilmenite (FeTiO_3_) itself, which additionally has been proven to adopt the related and technologically interesting LiNbO_3_ structure (space group *R*3*c*, Figure 1c) at high temperature and pressure [9]. In the *R*3*c* structure, the two different cations alternate perfectly, both parallel and perpendicular to the *c* axis. Distinguishing between the three different symmetries by the position of the reflections using X-ray diffraction is difficult. Unit cell values obtained from density functional theory calculations on *R*−3*c* and *R*3*c* are virtually the same [10], and effects from strain or off-stoichiometry are likely to be much larger. However, determining whether the symmetry is *R*−3 or *R*−3*c*/*R*3*c* is easy, as the diffraction pattern from *R*−3 has reflections from ordering not allowed with the *c* glide plane of *R*−3*c*/*R*3*c* due to extinction rules. While there are no reports of bulk NTO with *R*3*c* structure, the phase was claimed to have been obtained by Varga et. al on α-Al_2_O_3_ substrates as thin films made by pulsed-laser deposition [10]. Even though the symmetry was not unambiguously confirmed, some degree of lattice polarization was observed, and possibly weak ferromagnetism, indicating the presence of the LiNbO_3_ structure [11,12,13]. Ferroelectricity and weak ferromagnetism have indeed been observed in bulk samples of NTO as well [14], but the report did not discuss the crystal symmetry of the samples.

Thin films of NTO have previously been made by various deposition techniques, including aerosol-assisted CVD [2], sol-gel methods [3,15,16,17,18], dip-coating [19] and RF-sputtering [4,5], as well as with the previously mentioned pulsed laser deposition [10,11,12]. Especially for the latter technique, compositional control seems to be challenging. In addition, all methods require elevated temperatures (>500 °C) either during deposition or by post-deposition annealing. While amorphous films might be desirable for some applications, a well-defined crystallinity and orientation is usually required to make use of the material. The atomic layer deposition (ALD) technique offers an alternative route for deposition of epitaxial thin films, provided careful selection of substrates [20,21], at relatively low temperatures (typically <400 °C). We have previously reported details about the deposition of NTO by ALD [22]. As deposited films on Si(100) with a 1:1 ratio between Ni and Ti (within 1%, as measured by X-ray fluorescence), all showed a preferred (001) orientation in the deposition temperature range 175–275 °C. No sign of the ordering reflections belonging to the *R*−3 symmetry were visible at any temperature, neither before nor after annealing. Given the difficulty of obtaining the *R*3*c* phase in bulk, the films deposited on Si(100) were assumed to have the disordered *R*−3*c* symmetry. However, from the limited amount of data the *R*3*c* symmetry cannot be ruled out. In the work presented here, we show the possibility to control the orientation and crystallinity of deposited NTO films with the use of various single-crystal substrates. As with the previously reported films deposited on Si(100), the films discussed here are assumed to have the *R*−3*c* symmetry whenever the ordering reflections are not present. The ability to control the phase and orientation of these films is interesting with respect to catalysis. In addition, by using ALD it should be easy to incorporate other elements. Doping of NiTiO_3_ has already been shown to improve solar water splitting efficiency [23], as well as inducing ferromagnetism in coexistence with ferroelectricity [24].

## 2. Materials and Methods 

The films were deposited in an F-120 Sat reactor (ASM Microchemistry Ltd., Helsinki, Finland) using the precursors Ni(acac)_2_ (nickel acetylacetonate, 95%, Sigma-Aldrich, St. Louis, MO, USA) in combination with O_3_ (produced in a BMT Messtechnik GMBH ozone generator from 99.6% O_2_, AGA), and TTIP (Titanium(IV) tetraisopropoxide, 97%, Sigma-Aldrich, St. Louis, MO, USA) in combination with deionized water. A pulsing and purging sequence of (2 s Ni(acac)_2_–1.5 s purge–3 s O_3_–2 s purge) + (0.5 s TTIP–1 s purge–2 s H_2_O–3 s purge) were used for all depositions, as described more thoroughly in our prior work on deposition of NTO [22]. The substrates used were 3 × 3 cm^2^ single crystals of Si(100), for thickness and composition determination, as well as *α*-Al_2_O_3_(001) (referred to simply as Al_2_O_3_ from now on), LaAlO_3_(100) (LAO), SrTiO_3_(100) (STO) and MgO(100) for structural analysis. All dust was blown clean of substrates using pressurized air before being placed in the reaction chamber, and subjected to 15 s of O_3_ immediately before deposition. The films grown on Al_2_O_3_ and MgO were deposited in the same experiment with a nominal thickness of 165 nm (measured on Si(100) substrates). Likewise, the films grown on STO and LAO were deposited in the same experiment with a nominal thickness of 130 nm (measured on Si(100) substrates). All depositions were performed at 250 °C. Annealing was undertaken in air by rapid thermal processing (RTP) in an MTI Corporation OTF-1200X furnace (Richmond, VA, USA). The heating program consisted of a 15 min. ramp from room temperature to 650 °C, followed by a dwell time of 15 min., and subsequent cooling in the furnace to room temperature, for a typical duration of 30 min.

The film thicknesses were measured by spectroscopic ellipsometry on Si(100) substrates, using a J.A. Woollam (Lincoln, Dearborn, MI, USA) alpha-SE ellipsometer. The CompleteEASE software package (version 4.92, J.A. Woollam, Lincoln, Dearborn, MI, USA) was used to fit a Cauchy function to the obtained data in the 390–900 nm wavelength range. Measurements on several positions on the 3∙3 cm^2^ Si substrates were averaged (std.dev. ~3 nm) used to indicate a “nominal thickness” for the films on single crystalline oxide substrates. The cationic composition was determined by X-ray fluorescence (XRF) measurements on the Si substrates using a Philips (Almelo, The Netherlands) PW2400 spectrometer and the UniQuant analysis software (version 2, Omega Data Systems, Veldhoven, The Netherlands). All *θ*-2*θ* X-ray diffraction (XRD) was performed on a Bruker AXS (Karlsruhe, Germany) D8 Discover diffractometer in Bragg–Brentano configuration with Cu K*α* radiation. The diffractometer was equipped with a Ge(111) monochromator and a LynxEye strip detector. Reciprocal space maps and *φ* scans were collected using a PANalytical Empyrean diffractometer, equipped with a Cu K*α* source powered at 45 kV/40 mA, a hybrid monochromator and a PIXcel^3D^ detector (PANalytical, Almelo, The Netherlands). Atomic force microscopy (AFM) was performed with a Park Systems (Santa Clara, CA, USA) XE-70 AFM, equipped with a PPP-CONSTCR tip (Nanosensors, Neuchâtel, Switzerland) in contact mode.

Literature values for NTO in this paper refer to the PDF# 01-075-3757, ICDD. The listed cell parameters of the unit cell, having the ilmenite structure (*R*−3), are *a* = 5.0321 Å and *c* = 13.7924 Å.

## 3. Results

To provoke different crystallographic orientations, films were deposited on a range of single crystalline substrates: Al_2_O_3_(001), LAO(100), STO(100) and MgO(100). The results will be presented in this order below.

### 3.1. On Al_2_O_3_(001)

An obvious choice of substrate for growth of NTO thin films is Al_2_O_3_, as the two materials adopt closely related crystal structures. Al_2_O_3_ lends its mineral name to the corundum structure (*R*−3*c*), while NiTiO_3_ has the same structure as its iron counterpart ilmenite (FeTiO_3_), at room temperature (*R*−3) (Figure 1). The unit cell parameters of Al_2_O_3_ (*a* = 4.76 Å, *c* = 12.99 Å, PDF# 00-046-1212, ICDD ) are distinctly smaller than for NTO (*a* = 5.03 Å, *c* = 13.79 Å, PDF# 01-075-3757, ICDD).

**Figure 2:**
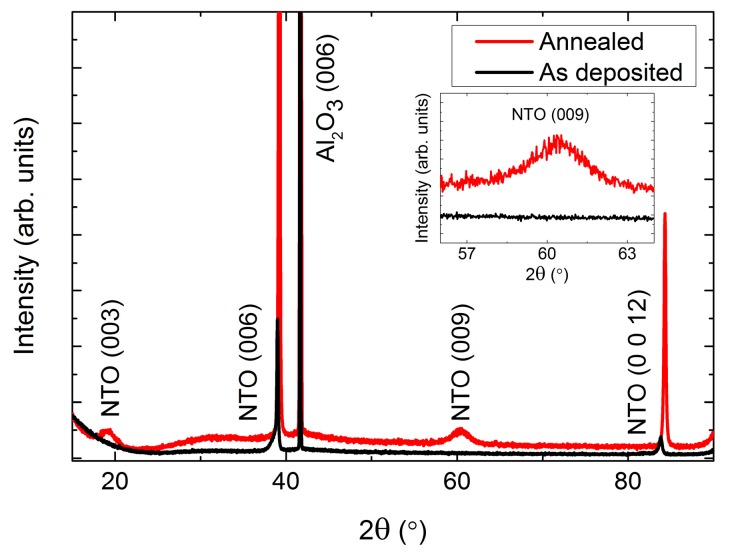
X-ray diffraction (XRD) of (00*l*) oriented NiTiO_3_ (NTO) on Al_2_O_3_(001) showing the transition from space group *R*−3*c* as deposited (**black line**) to *R*−3, upon annealing (**red line**). The ordering reflections (003) and (009) are not visible for the as deposited films, as shown in the inset.

As-deposited NTO films were (00*l*) oriented on Al_2_O_3_(001), as observed by *θ*-2*θ* X-ray diffraction (Figure 2). Upon annealing, two additional reflections appeared: (003) and (009). These reflections are only present for the *R*−3 symmetry due to extinction rules, and can thus be used to differentiate between cation order (*R*−3) and disorder (*R*−3*c*). It should be noted that the (003) and (009) reflections are also absent for the *R*3*c* symmetry. However, as mentioned in the introduction, since this symmetry has never been irrefutably observed for NTO it is assumed in the following that the (00*l*) oriented films presented here have the *R*−3*c* symmetry, whenever the (003) and (009) reflections are not present.

**Figure 3:**
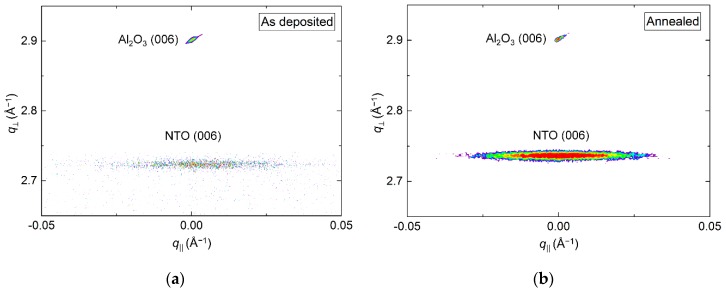
Reciprocal space map (RSM) of the symmetrical (006) reflections from NTO and Al_2_O_3_ as deposited (**a**) and after annealing (**b**).

The crystallinity of the film improved drastically upon annealing, clearly visible from the reciprocal space map (RSM) of the NTO (006) reflection by an increase in intensity (Figure 3). A decrease in full width at half maximum (FWHM) along *q*_‖_, from 1.0° to 0.7°, was also observed. Annealing shortened the *c* axis length, visible as a shift of the position of the (006) reflection to a larger *q* value, along *q*_⏊_. The *c* axis was 0.4% longer in the as deposited film and 0.1% shorter in the annealed film, compared to the literature value.

**Figure 4:**
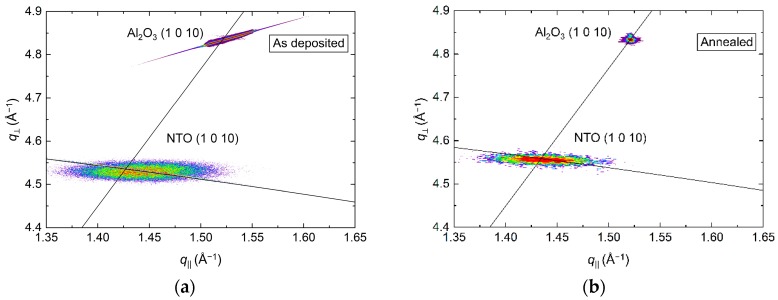
RSM of the asymmetrical (1 0 10) reflections from NTO and Al_2_O_3_ before (**a**) and after (**b**) annealing. The arced lines illustrate the Ewald sphere, while the straight lines are a guide to the eye that intersects the origin and the Al_2_O_3_ (1 0 10) reflection.

RSM of the (1 0 10) asymmetrical reflections in Figure 4 show the in-plane relaxation of the film upon annealing. For the as deposited film, the position of the NTO (1 0 10) reflection indicated an *a* axis 0.1% shorter than the literature value. Annealing resulted in a 0.1% longer *a* axis, again, compared to the literature value. The FWHM along the Ewald sphere was also reduced upon annealing, from 1.2° to 1.0°.

**Figure 5:**
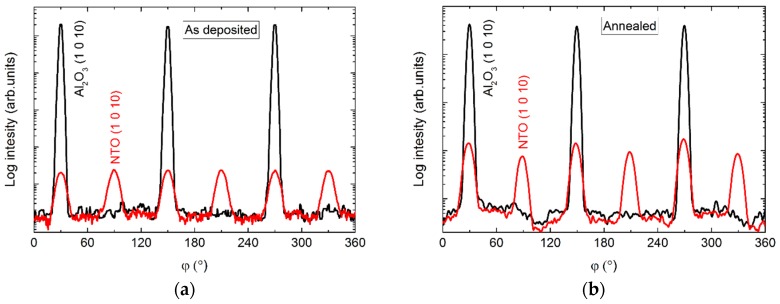
*φ* scan of the (1 0 10) reflections from NTO (**red lines**) and Al_2_O_3_ (**black lines**) as deposited (**a**) and after annealing (**b**).

The *φ* scans of the (1 0 10) reflection revealed a six-fold rotational symmetry (Figure 5), with three of the reflections overlapping those from the substrate, and the other three shifted by 60°.

**Figure 6:**
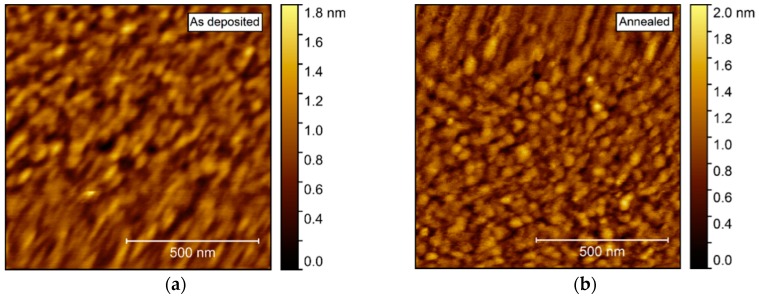
Atomic force microscopy (AFM) images of NTO deposited on Al_2_O_3_(001). As deposited root mean square (rms) = 0.21 nm (**a**), increasing to rms = 0.24 nm after annealing (**b**).

AFM investigations of the as deposited films showed a very flat surface, with a root mean square (rms) roughness of only 0.21 nm (Figure 6). After annealing, the roughness increased only slightly, to 0.24 nm. No recognizable facets could be identified, but rather many smaller, round crystallites, possibly convoluted by the AFM tip itself.

### 3.2. On LaAlO_3_(100)

LAO has a distorted perovskite crystal structure, with space group symmetry *R*−3*c*, the same as the high temperature disordered phase of NTO. Compared to NTO, LAO (PDF 00-031-0022, ICDD) has a longer *a* axis (5.36 Å vs. 5.03 Å), but a shorter *c* axis (13.11 Å vs. 13.79 Å). However, LAO can also be represented as a pseudocubic structure, with angles that are less than 0.1° off from 90°. In this regard, the unit cell space group is *Pm*−3*m*, and *a* = 3.79 Å. From here comes the given orientation of the substrates used in this work: LAO(100), which is the same as LAO(012) in the rhombohedral system. The orientation and reflections of LAO in this text refer to the pseudocubic system, unless otherwise stated. There is no pseudocubic symmetry for NTO similar to LAO, given the same orientation as for LAO, with pseudocubic(100) = rhombohedral(012). The closest equivalent is a unit cell with dimensions *a* = 5.44 Å; *b* = 5.03 Å; *c* = 3.66 Å, and angles *α* = 90°; *β* = 83.1°; *γ* = 90°.

**Figure 7:**
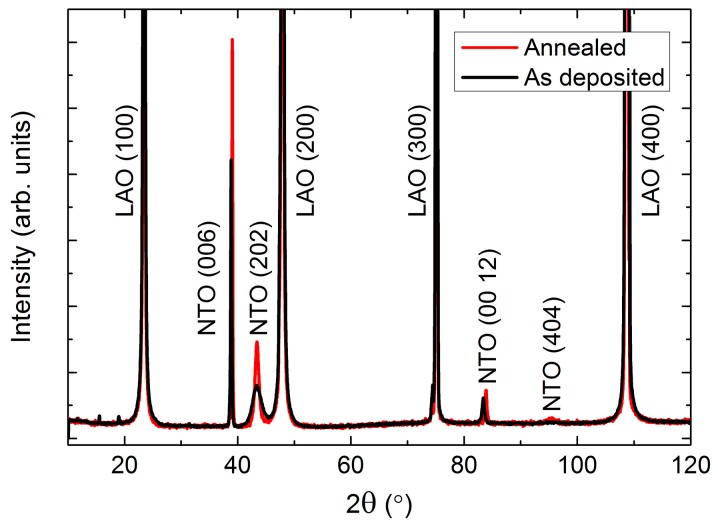
XRD of NTO deposited on LaAlO_3_(100) (LAO(100)), as deposited (**black line**) and after annealing (**red line**).

NTO films deposited on LAO(100) substrates had two preferred orientations: (00*l*) and (*h*0*h*) (Figure 7). Reflections from the (*h*0*h*) orientation were considerably weaker and broader than from the (00*l*) orientation. In addition, annealing increased the intensity and reduced the FWHM for all reflections, with (*h*0*h*) reflections remaining distinctly broader.

**Figure 8:**
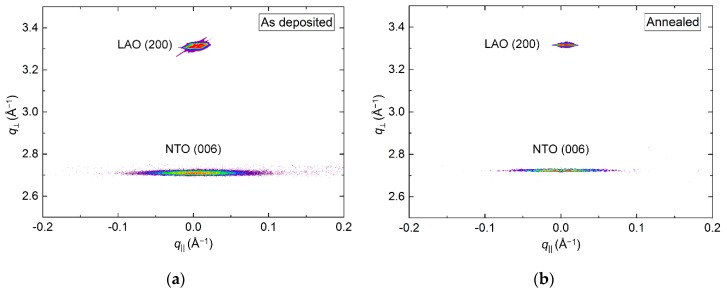
RSM of the symmetrical (006) reflection from NTO along with the LAO (200) reflection, before (**a**) and after (**b**) annealing.

RSM of the (006) reflection revealed a small shift in *q*_⏊_ direction upon annealing, with the length of the *c* axis being 0.8% and 0.2% larger than the literature value before and after annealing, respectively (Figure 8). Annealing also reduced the FWHM from 2.3° to 2.1°, along *q*_‖_. Extensive efforts were made to collect decent RSMs of the (*h*0*h*) reflection, but with no success. This was also the case for any related asymmetrical reflections.

**Figure 9:**
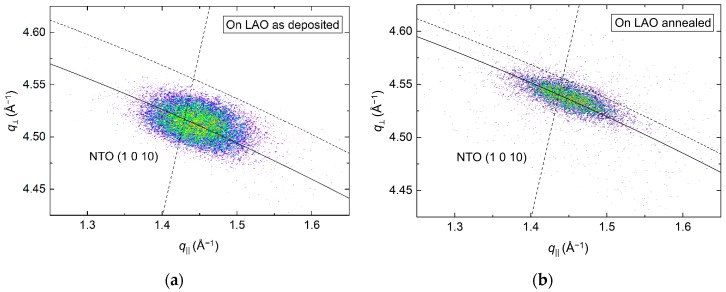
RSM of the asymmetrical NTO (1 0 10) reflection from a film deposited on LAO(100), as deposited (**a**) and after annealing (**b**). The solid arced lines illustrate the curvature of the Ewald sphere. The intersection of the dashed lines mark the theoretical position of the NTO (1 0 10) reflection.

From the (00*l*) related asymmetrical reflection (1 0 10) in Figure 9, the *a* axis was calculated to be 0.3% and 0.5% shorter compared to the literature value before and after annealing, respectively. The shift along *q*_⏊_ corresponded well with the compression of the *c* axis upon annealing, and a distinct reduction of the FWHM along the Ewald sphere from 1.5° to 1.1° was also observed.

**Figure 10:**
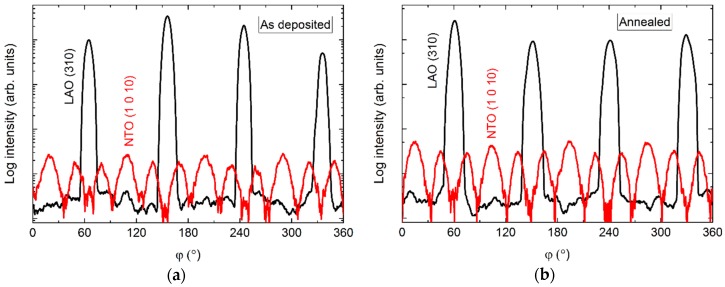
*φ* scans of the NTO (1 0 10) reflection (**red line**) along with the LAO(310) reflection (**black line**), before (**a**) and after (**b**) annealing.

The *φ* scans revealed a 12-fold symmetry out-of-phase with the four-fold symmetry of the selected substrate reflection (Figure 10). Annealing did not affect the symmetry, and the only observable change was a slight increase in intensity.

**Figure 11:**
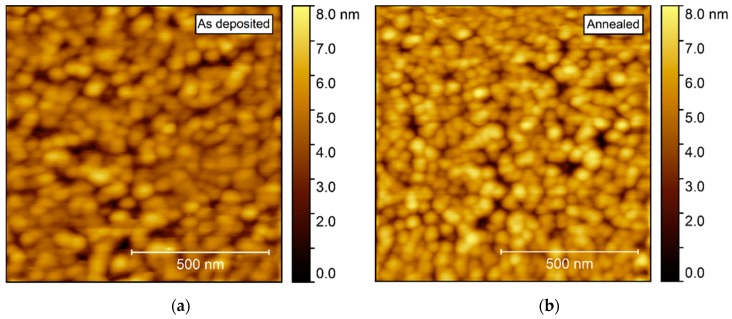
AFM images of NTO deposited on LAO(100). As deposited rms roughness = 0.9 nm (**a**). Annealed rms roughness = 1.0 nm (**b**).

Annealing induced no apparent change in the topography, as observed by AFM (Figure 11). The surface was comprised of spherical-like crystallites, similar to the films deposited on Al_2_O_3_, but with a larger rms roughness, at 0.9–1.0 nm.

### 3.3. On SrTiO_3_(100)

The pseudocubic representation of LAO is closely related to STO, a perovskite structure with *Pm*3*m* symmetry. The cell parameters of STO are slightly larger compared to LAO, with *a* = 3.90 Å (PDF 00-035-0734, ICDD).

**Figure 12:**
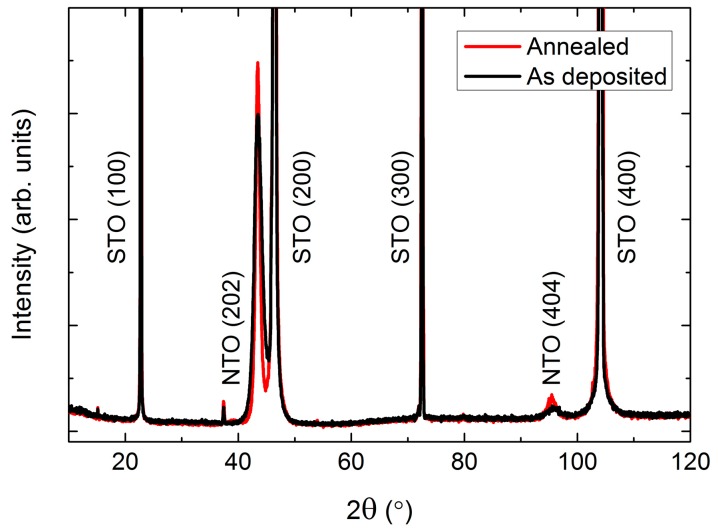
XRD of (*h*0*h*) oriented NTO deposited on SrTiO_3_(100) (STO(100)). A slight increase in intensity was observed after annealing (**red line**), compared to the as deposited film (**black line**).

Depositions on STO(100) resulted in (*h*0*h*) orientated NTO (Figure 12). The intensity of the reflections were higher compared to the same reflections on LAO, but the relative increase upon annealing was smaller, indicating an initial higher crystallinity of the as deposited film. The FWHM values were very similar for the two systems, both as deposited and after annealing.

**Figure 13:**
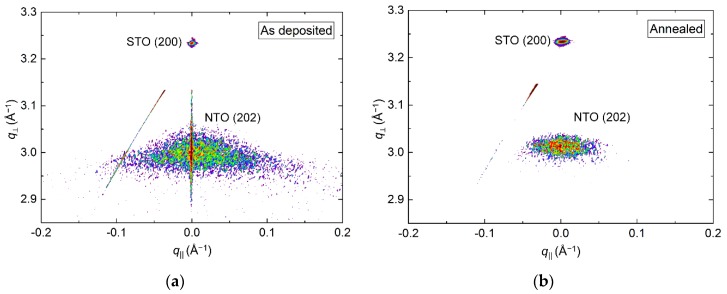
RSM of the symmetrical NTO(202) reflection along with STO(200) before (**a**) and after (**b**) annealing. Detector streaking is visible in both RSMs, with the as deposited data also showing wavelength streaking through the film reflection.

RSM of the symmetrical (202) reflection of as deposited and annealed films revealed a reduction in the FWHM from 3.4° to 2.5° along *q*_‖_ upon annealing (Figure 13). A small shift in the *q*_⏊_ direction was also observed, with the calculated *c* axis being 0.8% (as deposited) and 0.5% (annealed) larger than the literature value.

**Figure 14:**
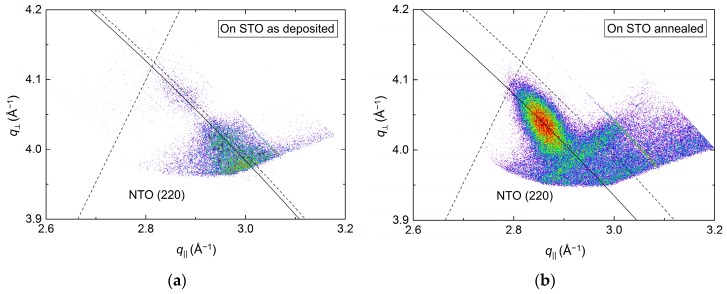
RSM of the asymmetrical (220) reflection from NTO on STO(100) as deposited (**a**) and after annealing (**b**). The solid arced lines illustrate the curvature of the Ewald sphere. The intersection of the dashed lines mark the theoretical position of the NTO(220) reflection.

Conversely, investigations of the asymmetrical reflection showed a clear shift along the Ewald sphere (Figure 14), indicating a pronounced relaxation of the structure. However, a low-intensity reflection closer to the relaxed position was visible for the as-deposited film as well, indicating that the initial strain did not persist throughout the as-deposited film. A reduction in FWHM along the Ewald sphere was observed upon annealing, from 1.6° to 0.8°. As the NTO(220) reflection was very close to the edge of the accessible region of the instrument, and possibly out of reach for the as-deposited film, these values are very uncertain. This is also reflected in the calculated *a* axis values. Compared to literature, the *a* axis was 5.0% and 1.5% smaller for the as deposited and annealed films, respectively.

**Figure 15:**
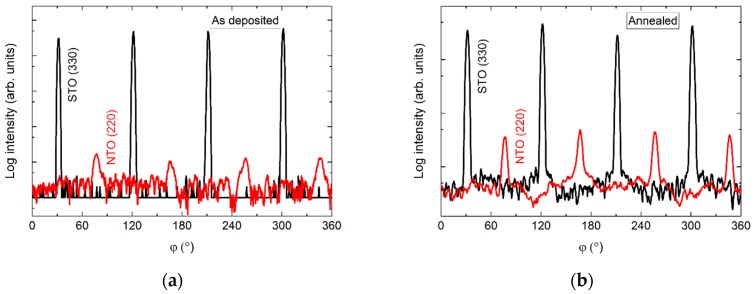
*φ* scans of the (220) reflection from NTO (red line) superimposed on the *φ* scan of the STO(330) reflection (black line) before (**a**) and after annealing (**b**).

The *φ* scan of the asymmetrical (220) reflection displayed a four-fold symmetry out-of-phase with the selected asymmetrical reflection from the substrate (Figure 15). Again, a slight increase in intensity was observed upon annealing.

**Figure 16:**
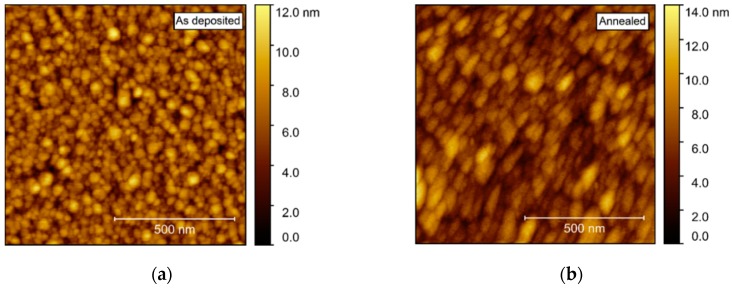
AFM images of NTO on STO(100), with rms roughness of 1.5 nm as deposited (**a**) and 1.7 nm after annealing (**b**).

Compared to films deposited on Al_2_O_3_, AFM of films deposited on STO revealed a considerably higher rms roughness, increasing from 1.5 to 1.7 nm upon annealing (Figure 16). As for the films deposited on Al_2_O_3_, the surface consisted of numerous round crystallites with no recognizable facets.

### 3.4. On MgO(100)

**Figure 17:**
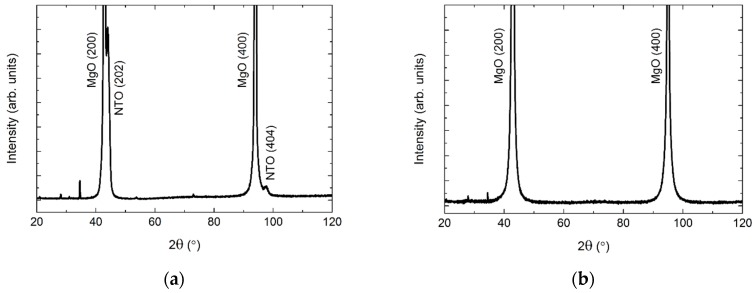
XRD of NTO on MgO(100) (**a**), showing the (*h*0*h*) orientation of the film. A diffractogram of an uncoated MgO(100) substrate is shown in (**b**) for comparison. Minor substrate artefacts are visible at 2*θ* = 28°, 34°, 54° and 73°.

MgO also has a cubic structure (*Fm*−3*m*), but with larger unit cell parameters (*a* = 4.21 Å, PDF 00-045-0946, ICDD) as compared to STO. As on STO, the deposited film was (*h*0*h*) oriented (Figure 17). Since the *d* spacing of NTO(*h*0*h*) and MgO(*h*00) are almost the same, a diffractogram of the pure MgO substrate is shown in Figure 17.

**Figure 18:**
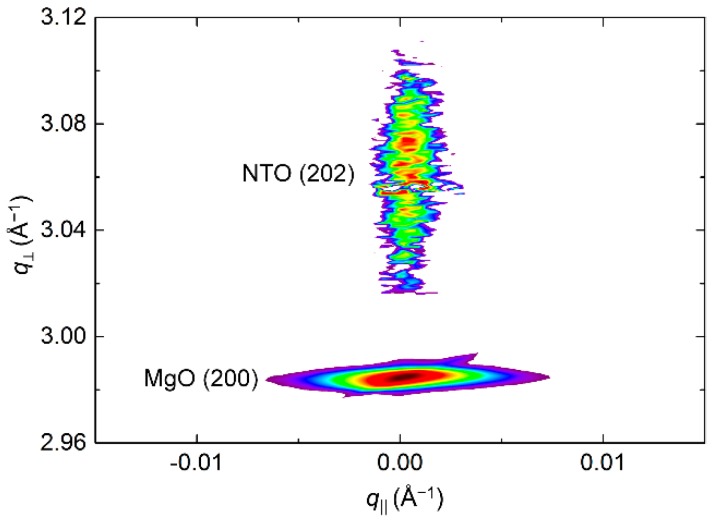
RSM of the symmetrical NTO (202) reflection as deposited, along with the MgO(200) reflection.

RSM of the symmetrical (202) reflection disclosed a very sharp peak with FWHM along *q*_‖_ of only 0.2° (Figure 18). The position of the reflection along *q*_⏊_ corresponds to a unit cell with a calculated *c* axis 1.2% shorter than the literature value.

**Figure 19:**
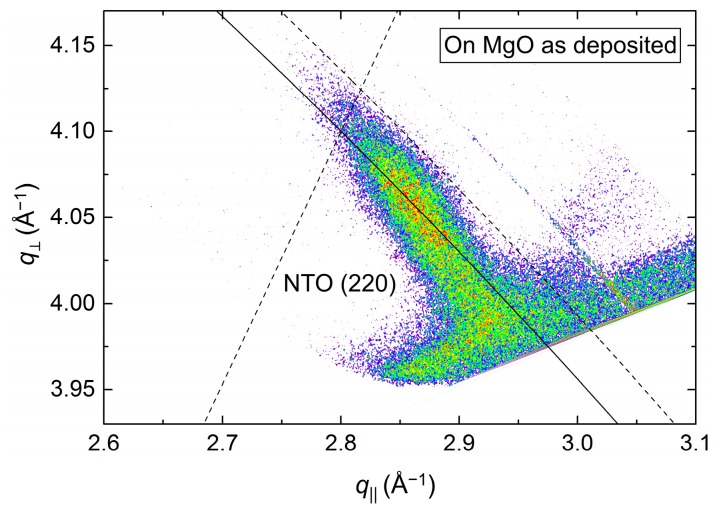
RSM of the asymmetrical NTO(220) reflection, as deposited on MgO(100). The Ewald sphere is illustrated as an arced solid line. The intersection of the dashed lines mark the theoretical position of the NTO(220) reflection.

The position of the asymmetrical reflection (220) (Figure 19) was close to that observed for the annealed film deposited on STO (assumed to be relaxed). The FWHM along the Ewald sphere, at 1.5°, was slightly smaller than for as-deposited NTO on STO. The calculated *a* axis was 1.2% shorter compared to the literature value.

**Figure 20:**
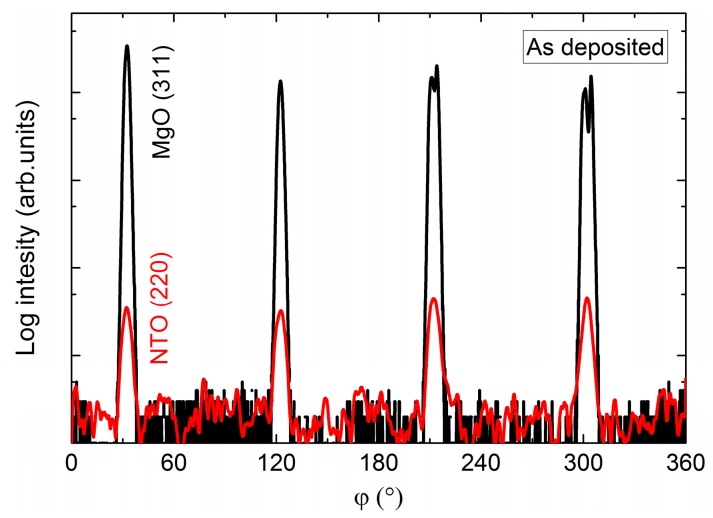
*φ* scan of the NTO(220) reflections (**red line**) superimposed on the *φ* scan of the MgO(311) reflections (**black line**).

In the same manner as for films on STO, the *φ* scan revealed a four-fold rotational symmetry of the film (Figure 20), but this time in-phase with the selected substrate reflection.

**Figure 21:**
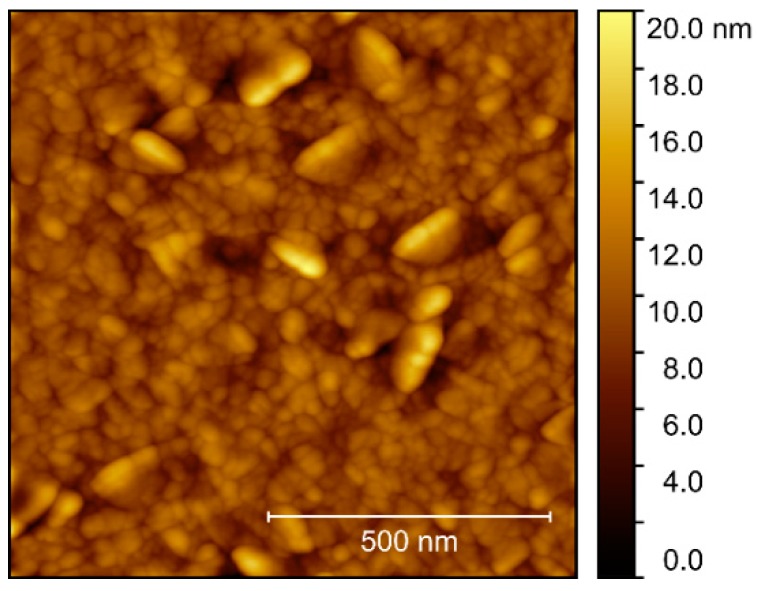
AFM image of NTO as deposited on MgO(100), with rms roughness = 1.9 nm.

Compared to the films deposited on other substrates, the surface topography of films grown on MgO was rather different. AFM scans revealed clear protrusions from a flatter bed of smaller crystals (Figure 21), with the flatter areas resembling the surfaces observed on the other substrates. The larger platelets, or flattened crystals, had no obvious collective orientation, and the rms roughness of the scanned area was 1.9 nm.

An overview of the results is given in Table 1. All the calculated cell parameters can be found in the Supplementary Material Section S1.

## 4. Discussion

### 4.1. On Al_2_O_3_(001)

Our previous report on the growth of NTO [22] showed that films deposited on Si(100) were (00*l*) oriented, with the orientation assumed to be from an inherent preferred growth direction. Annealing of those samples improved the crystallinity, but did not alter the symmetry, as seen by the absence of the (003) and (009) reflections. On Al_2_O_3_, the same *R*−3*c* symmetry is observed for as deposited films, but annealing induces ordering of the structure, resulting in the *R*−3 symmetry. For NTO, the disordered phase is only thermodynamically stable at temperatures above 1292 °C [7,8,25], and has not previously been obtained at room temperature by any other technique. The present case is thus yet another example of the ability of ALD to produce films with metastable phases, given careful selection of substrates and temperature treatment [26].

The mismatch factor, *f*, is usually given as *f* = 100% ∙ (*a_s_* − *a_f_*)/*a_s_*, where *a_s_* and *a_f_* denote the lattice parameters of the substrate and the film, respectively. The relevant way of looking at film-substrate mismatch in this system would be to compare the oxygen lattices at the interface. This gives a mismatch of −3.0%, when comparing the average O–O distances for the (001) surface of Al_2_O_3_ and NTO. The negative value of the mismatch indicates that there is a compressive strain from the substrate. Indeed, the as-deposited film has a shorter *a* axis, compared to the literature value. At the same time, the *c* axis is longer, due to the Poisson effect. Annealing relaxes the film, both in the basal plane and in the (00*l*) direction, with only 0.1% strain left.

The broadening of the symmetrical NTO (006) reflection along *q*_‖_ and the asymmetrical NTO (1 0 10) reflection along the Ewald sphere indicates that the film consists of numerous crystallites with somewhat random texture. This could stem from abundant nucleation early in the deposition, and that the numerous crystals grow as narrow pillars throughout the film. The resulting film would have many grain boundaries and a surface with many smaller crystallites, which consequentially results in a low roughness, as indeed observed by AFM.

**Figure 22:**
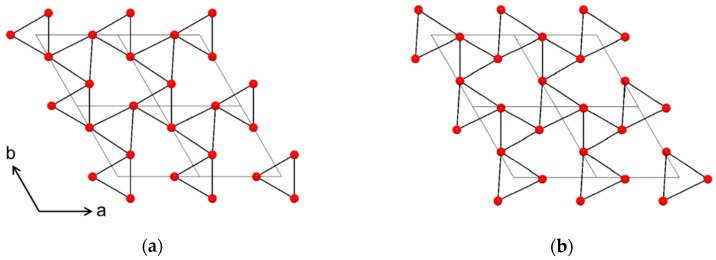
Oxygen lattices of the (001) plane in Al_2_O_3_, viewed along the *c* axis, showing the 60° rotation between the top (**a**) and bottom (**b**) faces of octahedrons around Al^3+^. The four grey diamonds in each cartoon indicate the edges of four unit cells.

With an epitaxial relationship between substrate and film, one might expect the same multiplicity of reflections in the *φ* scan. However, as presented above, the NTO film has twice the number of reflections compared to Al_2_O_3_. We hypothesize that this comes from atomic steps on the substrate surface revealing trigonal surface terminations rotated at 60° from each other (Figure 22), as also seen for growth of CaCO_3_ on Al_2_O_3_(001) [27]. The apparent six-fold rotational symmetry of the NTO film stems from two sets of crystallites, each with three-fold rotational symmetry, superimposed on each other. This feature also increases the likelihood of high nucleation density. The heteroepitaxial film is both out-of- and in-plane oriented, even though the number of reflections in the *φ* scan is different for the film and the substrate, resulting in the film||substrate epitaxial relationship: NTO(001)[100]||Al_2_O_3_(001)[100].

### 4.2. On LaAlO_3_(100)

While the epitaxial relationship for NTO||Al_2_O_3_ is easy to envision, the same is not true for NTO||LAO. There are no obvious lattice matches between either of the two preferred orientations of the deposited film and the (100) surface of the LAO substrate. In addition, it is hard to untangle the nature of the (*h*0*h*) orientation, as it was impossible to map any related asymmetrical reflections.

**Figure 23:**
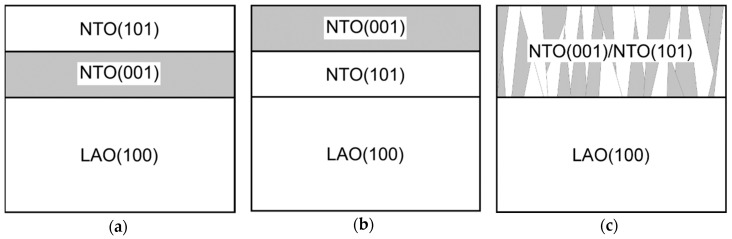
Cartoon of possible constellations for the two orientations observed in NTO on LAO.

With two preferred orientations the film can grow in one of three ways: either (a) the film consists of a layer with (00*l*) orientation close to the substrate and a (*h*0*h*) oriented layer on top, (b) the layers in (a) are inverted, or (c) there is a mix of crystallites with both orientations and no layered structure (Figure 23).

We are unfortunately unable to conclude which constellation is the most probable, even with basis in the observed variations in texture and surface roughness of the films obtained in this work (see Supplementary Material Section S2 for a more thorough discussion).

The 12 NTO (1 0 10) reflections observed in the *φ* scan can be explained similarly to NTO on Al_2_O_3_. That is, the film does not actually have a 12-fold rotational symmetry, but four sets of crystallites—each set in-plane oriented, with a three-fold rotational symmetry. The orientation of the sets of crystallites are shifted by 90° with respect to each other, resulting in 12 reflections in the *φ* scan. Both the substrate and a NTO(*h*0*h*) layer could facilitate this. In any case, it is clear that the nucleation density is high, as the different sets of NTO(00*l*) crystallites must have nucleated separately. If the NTO(00*l*) crystallites nucleated on the LAO(100) surface, as in constellation (a) or (c), the epitaxial relationship with the substrate would be: NTO(001)[100]||LAO(100)〈010〉, with 〈010〉 representing the four identical [010] directions on the LAO(100) surface.

### 4.3. On SrTiO_3_(100)

**Figure 24:**
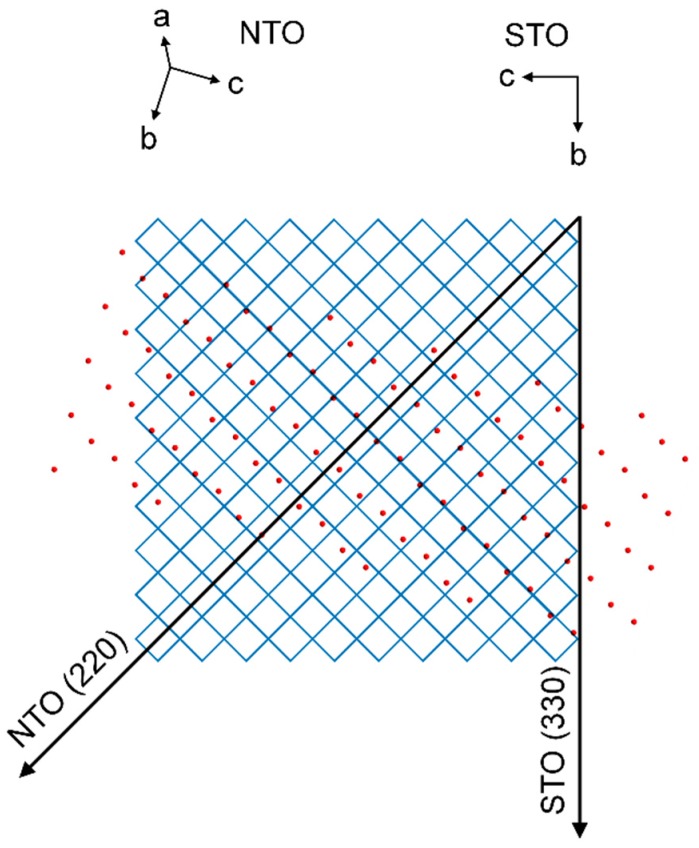
Schematic showing the direction of the STO (330) scattering vector projected down on the oxygen lattice of the STO(100) plane, represented by the corners of the blue matrix. Superimposed is the direction of the NTO (220) scattering vector projected down on the substrate plane, and the oxygen lattice of the NTO(101) plane, represented by red circles.

As with NTO on LAO, there are no obvious lattice matches between the film and the substrate. Still, the deposited film shows an in-plane (*h*0*h*) orientation. The four asymmetrical reflections from the film are perfectly out of phase with the four reflections from the substrate. This means that the direction of the NTO (220) scattering vector, projected down to the substrate surface, is 45° off from the same projection of the STO (330) scattering vector. Figure 24 shows the STO(100) and NTO(101) oxygen lattices, with the film lattice rotated according to the projections of the asymmetrical scattering vectors (see Supplementary Material Section S3 for details). The average O–O distance of the film lattice perpendicular to the projection of the NTO (220) direction is close to the oxygen spacing of STO(100). The mismatch along one row of NTO oxygen atoms is only −0.6%. However, when considering the direction along the NTO (220) scattering vector the mismatch between every second layer of oxygen atoms in STO(100) compared to every layer in NTO(101) is 18.4%.

The number of reflections in the *φ* scan can be explained in the same way as for NTO on Al_2_O_3_ and LAO. A single crystalline, (*h*0*h*) oriented, NTO film would have only one (*hh*0) reflection in a *φ* scan. The STO(100) surface has four identical (010) directions: (010), (0$\bar{1}$0). (001) and (00$\bar{1}$), resulting in the four-fold rotational symmetry seen in the *φ* scan. As the NTO film does not nucleate as a continuous layer, the initial islands of (*h*0*h*) oriented crystallites do not differentiate between the STO〈010〉 directions. Some of the crystallites will be in-plane oriented with the STO(010) direction, and some with the other directions. An epitaxial relationship between film and substrate can thus be described as: NTO(101)[220]||STO(100)〈010〉.

### 4.4. On MgO(100)

**Figure 25:**
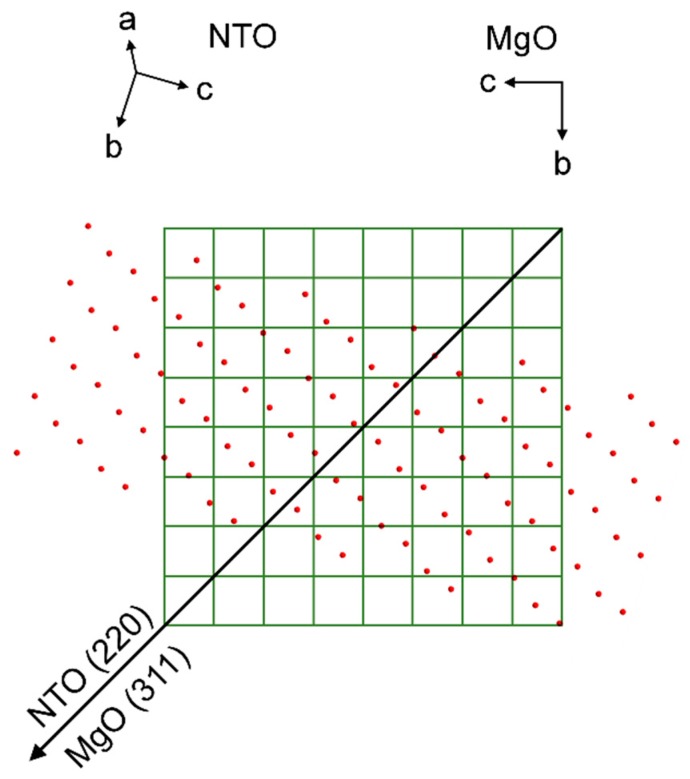
Schematic showing the direction of the MgO (311) scattering vector projected down on the oxygen lattice of the MgO(100) plane, represented by the corners of the green matrix, which also corresponds to the unit cell of MgO. Superimposed is the direction of the NTO (220) scattering vector projected down on the substrate plane, and the oxygen lattice of the NTO(202) plane, represented by red circles.

Unlike the previous cases, AFM of NTO on MgO show clear signs of bigger crystals. This resonates well with the smaller FWHM of the symmetrical NTO (202) reflection, indicating the presence of larger and/or less tilted crystallites. However, again, there are no obvious lattice matches between the MgO(100) surface and NTO, neither in general, nor when comparing the NTO(101) oxygen lattice with the oxygen lattice of the MgO(100) surface. Nevertheless, the film is indeed in-plane orientated with the substrate, but in the same manner as for NTO on STO. The φ scan reflections from the substrate and film are overlapping. This means that the direction of the asymmetrical scattering vectors, when projected down on the MgO(100) surface plane, are pointing in the same direction (Figure 25). Regarding a row of NTO oxygen atoms perpendicular to the projection of the MgO (311) scattering direction, there is a seemingly good lattice match for every second layer of oxygen atoms. The calculated mismatch is only 2.4%, but as can be seen from Figure 25, this is only true for selected rows of NTO oxygen atoms. When considering the whole oxygen lattice of NTO it does not correspond very well with the MgO(100) oxygen lattice. Still, the film is in-plane oriented with all the identical MgO〈011〉 directions, and the epitaxial relationship is determined to be: NTO(101)[220]||MgO(100)〈011〉.

## 5. Conclusions

Oriented NTO films were successfully deposited at 250 °C by ALD on various single crystalline substrates. All films had preferred orientations as deposited. Crystallinity improved after annealing at 650 °C for 15 minutes, but did not change the preferred orientations. On Al_2_O_3_(001), annealing led to cation ordering, and a change of symmetry from *R*−3*c* to *R*−3, visible by the appearance of the (003) and (009) reflections. However, the intensity was much lower, and the FWHM much larger, than for the other (00*l*) reflections, indicating that only parts of the film underwent cation ordering. AFM revealed very low rms roughnesses of about 0.2 nm, both for the as deposited and annealed film. The film||substrate epitaxial relationship was determined to be NTO(001)[100]||Al_2_O_3_(001)[100]. Depositions on LAO(100) resulted in films with two preferred orientations: NTO(*h*0*h*) and NTO(00*l*). There were no signs of ordering reflections for either orientation, indicating a *R*−3*c* symmetry. Annealing improved the crystallinity slightly, but did not alter the orientation of the film, the cation ordering nor change the rms roughness significantly, remaining at approximately 1 nm. Films deposited on STO(100) had a NTO(101)[220]||STO(100)〈010〉 epitaxial relationship with the substrate, with no signs or ordering reflections Annealing resulted in a slight improvement of crystallinity and increase in roughness, from 1.5 to 1.7 nm. Films deposited on MgO(100) had *R*−3*c* symmetry and the film||substrate epitaxial relationship was NTO(101)[220]||MgO(100)〈011〉. Reciprocal space mapping of the symmetrical NTO (202) reflection revealed a very small FWHM along *q*_‖_, indicating large crystallites in the film, and/or a very low tilt of the crystallites. No annealing was performed, but AFM investigations displayed larger crystallites protruding from a flatter surface, and the rms roughness was significantly larger than for films on other substrates, at 1.9 nm.

## Figures and Tables

**Table 1:** Summary of results for NTO films deposited on various single crystal substrates. AD and Ann refers to as deposited and annealed samples, respectively. The *c* and *a* values were calculated from the positions of the symmetrical and asymmetrical reflections in the RSMs, respectively, and are relative to the literature values. The *ω*_FWHM_ values are the full width at half maximum of Gauss functions fitted to the RSMs of symmetrical reflections along *q*_‖_. The *ES*_FWHM_ values are the full width at half maximum of Gauss functions fitted to the asymmetrical reflections along the Ewald sphere. The *φ* values are the number of film reflections in the *φ* scans. Root mean square (rms) roughness values are from the built in analysis tool in the Gwyddion software.

Substrate	Film	State	*c* (%)	*ω*_FWHM_ (°)	*a* (%)	*ES*_FWHM_ (°)	*φ* Refl.	rms (nm)
Al_2_O_3_(001)	(00*l*)	AD	0.36	1.03	−0.13	1.16	6	0.21
		Ann	−0.11	0.67	0.09	0.96		0.24
LAO(100)	(00*l*)	AD	0.81	2.26	−0.29	1.49	12	0.90
		Ann	0.24	2.10	−0.45	1.06		0.96
STO(100)	(*h*0*h*)	AD	0.84	3.41	−5.01	1.61	4	1.50
		Ann	0.47	2.50	−1.52	0.77		1.71
MgO(100)	(*h*0*h*)	AD	−1.22	0.22	−1.20	1.46	4	1.93

## Supplementary Materials

Supplementary material is available online at https://www.mdpi.com/1996-1944/13/1/112/s1.
