# Supplementary material for: Phase and Orientation Control of NiTiO3 Thin Films
Source: Materials (Basel). 2019 Dec 25;13(1):112. doi: 10.3390/ma13010112 (PMC6982290; doi:10.3390/ma13010112)
Supplement: Supplementary file 1 [file materials-13-00112-s001.pdf]

materials

Supplementary Materials

Phase and Orientation Control of $NiTiO_3$ Thin Films

Jon Einar Bratvold *, Helmer Fjellvåg and Ola Nilsen

Centre for Materials Science and Nanotechnology (SMN), Department of Chemistry, University of Oslo, P.O. Box 1033 Blindern, N-0315 Oslo, Norway; helmer.fjellvag@kjemi.uio.no (H.F.); olnilsen@kjemi.uio.no (O.N.)
* Correspondence: j.e.bratvold@kjemi.uio.no

Received: 26 November 2019; Accepted: 20 December 2019; Published: date

Table S1: Calculated cell parameters from reciprocal space maps.

Substrate	Film Orient.	State	*c* (Å)	*a* (Å)
Al_2O_3(001)	(00l)	AD	13.842	5.026
		Ann	13.777	5.037
LAO(100)	(00l)	AD	13.905	5.017
		Ann	13.826	5.009
STO(100)	(h0h)	AD	13.908	4.780
		Ann	13.857	4.956
MgO(100)	(h0h)	AD	13.624	4.972

Section S2: $NiTiO_3$ (NTO) on $LaAlO_3$ (LAO)

From the FWHM (along $q_{\parallel}$) of the symmetrical reflections it is clear that the (006) reflection is significantly broader than for NTO on Al_2O_3, while the (202) reflection is slightly broader than for NTO on STO. This means that both orientations have smaller crystallites and/or that the crystallites are tilted more than for the respective films with only one orientation. The surface roughness value for NTO on LAO is halfway between the values for films deposited on Al_2O_3 and STO. For comparison, the two different orientations are assumed to have similar surface roughnesses as on other substrates, regardless of the layer beneath. The roughness value of NTO on LAO should then be larger than what is observed if the film is (*h*0*h*) terminated. Conversely, if the film surface is (00*l*) terminated, a lower roughness value is expected. However, for crystalline films, the surface roughness is usually also dependent on the film thickness. That is, a thicker, crystalline, film will have a higher roughness than a thinner one. In this respect, the film on LAO could very well have constellation (a). The films on LAO and STO are from the same deposition and hence their overall thickness is the same. A terminating (*h*0*h*) layer would, therefore, have to be thinner than the film on STO. However, the surface roughness of crystalline ALD films is indeed affected by the layer beneath, and the assumption above is, thus, void. If the film has constellation (b), the terminating (00*l*) layer grows on a much rougher surface than a polished substrate. This is expected to yield a higher roughness than for NTO on Al_2O_3. Finally, constellation (c) might also be probable, as the roughness value is almost exactly between the values found for films on STO and Al_2O_3. Without more information on the (*h*0*h*) orientation, it is hard to determine which of the proposed constellations the film actually has.

Section S3: In-Plane Orientation

Finding the in-plane orientation of the deposited films is achieved through simple geometry. The directions of the asymmetrical reflections for the substrates are found by disregarding the out-of-plane component (see Figures 24 and 25 in main article). The in-plane orientation of the films are found by orienting the unit cell so that the planes giving rise to symmetrical reflections are parallel with the substrate surface, and then rotating the unit cell according to the φ scans (see Figures 15 and 20 in the main article). To do this we first need the height of the basal plane of the unit cell. The basal plane can be divided into two equilateral triangles, together forming a rhombohedron. The height of these triangles, as shown in Figure S1, can be found using theoretical cell parameters for the $NiTiO_3$ (NTO) unit cell with Equation (1).

$$h = b \cdot \sin 60° = 5.03\ \text{Å} \cdot sin60° = 4.36\ \text{Å} \quad (1)$$

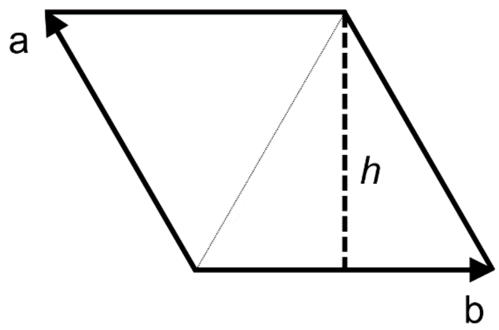

Figure S1: Rhombohedral unit cell viewed along the *c* axis, with the height of the basal plane of the unit cell marked as *h*. Note that the height intersects at the midpoint on the *b* axis.

Orienting the NTO($h0h$) planes parallel to the substrate surface is done by fixing the b axis of the NTO unit cell along the b axis of the substrate unit cell and tilting the NTO unit cell (Figure S2). The angle of this tilt is given by Equation (2).

$$\alpha = \text{atan}\frac{h}{c} = \text{atan}\frac{4.36\ \text{Å}}{13.79\ \text{Å}} = 17.55° \quad (2)$$

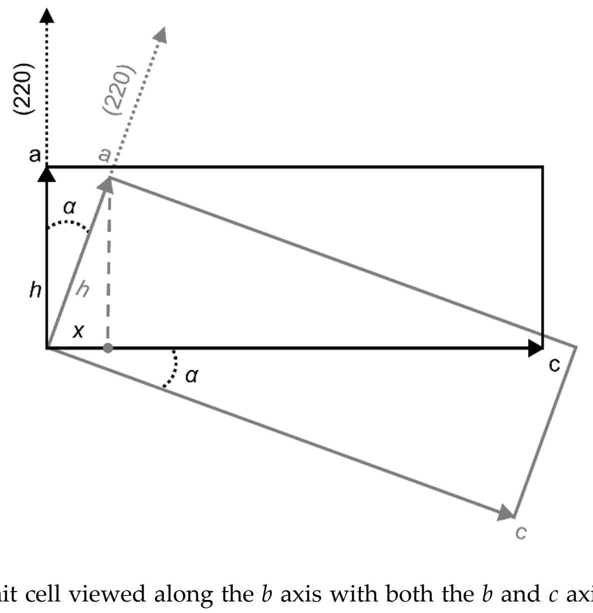

Figure 2. The NTO unit cell viewed along the *b* axis with both the *b* and *c* axis parallel to substrate surface (**black**). and after tilting (**grey**) to align the NTO(101) plane parallel to the substrate surface.

Next, the angle α from Equation (2) is used to find the x in Figure S2 by Equation (3):

$$x = h \cdot \sin\alpha = 4.36\ \text{Å} \cdot \sin 17.55° = 1.31\ \text{Å} \tag{3}$$

Lastly, by projecting the direction of the asymmetrical NTO scattering vector down on to the substrate surface (Figure S3) the angle of rotation β, relative to the b axis of the substrate, is found from Equation (4):

$$\beta = \text{atan}\frac{x}{\frac{b}{2}} = \text{atan}\frac{1.31\ \text{Å}}{2.515\ \text{Å}} = 27.51° \tag{4}$$

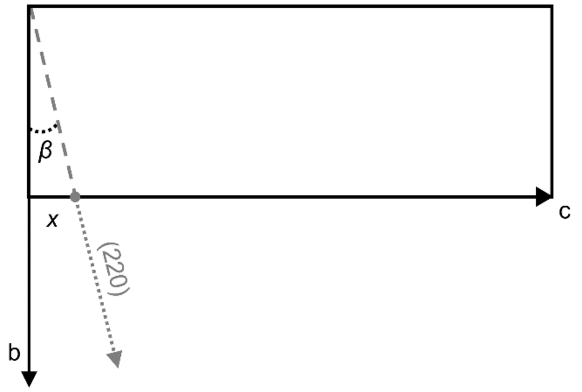

Figure 3. Direction of the NTO(220) scattering vector projected down onto the substrate surface. The unit cell at the starting point is marked out in black as a reference point, with the film *b* axis having the same direction as the substrate *b* axis.

The angle β can now be used to rotate a layer of the NTO(101) oxygen lattice in-plane to align it according to the φ scan. For the NTO||STO case, the investigated asymmetrical reflections lie 45° apart. This means that the oxygen lattice needs to be rotated 17.5° (45°–27.5°), to obtain a 45° separation between the NTO(220) and STO(330) scattering vector projections. In the NTO||MgO case, the projected scattering vectors have the same rotational direction, but the projected direction of the MgO(311) reflection is 45° off from the *b* axis. Thus, the rotation of the NTO(101) oxygen lattice is the same as for NTO on STO.

© 2019 by the authors. Submitted for possible open access publication under the terms and conditions of the Creative Commons Attribution (CC BY) license (http://creativecommons.org/licenses/by/4.0/).
